# Individualized acromegaly treatment: Is stereotactic radiation therapy changing the paradigm?

**DOI:** 10.3389/fendo.2022.1034576

**Published:** 2022-12-15

**Authors:** Winnie Liu, Maria Fleseriu

**Affiliations:** ^1^ Department of Medicine, Division of Endocrinology, Diabetes and Clinical Nutrition, Oregon Health & Science University, Portland, OR, United States; ^2^ Department of Neurological Surgery, Oregon Health & Science University, Portland, OR, United States; ^3^ Pituitary Center, Oregon Health & Science University, Portland, OR, United States

**Keywords:** acromegaly, stereotactic radiation, treatment, therapy, individualized

## Introduction

Acromegaly is rare disease of growth hormone (GH) excess, caused predominantly by somatotroph adenomas. Incidence ranges between 0.2−1.1 cases/100,000 people; however, the disease is likely underdiagnosed. Median diagnosis is in the fifth decade of life with at least a 4 to 5-year delay (1, 2), despite several common presenting features (2, 3).

Untreated and uncontrolled acromegaly leads to cardiovascular, respiratory, metabolic and neoplastic complications (4) and higher mortality rates vs. the general population (4, 5). As a result of improved life expectancy, cancer is now leading cause of death instead of cardiopulmonary complications based on a 2018 meta-analysis (6).

Improved patient outcomes are likely influenced by advanced treatment modalities including surgery, new pharmacological agents/regimens (single or in combination), somatostatin receptor ligands (SRLs; octreotide, lanreotide, and pasireotide), dopamine agonists (cabergoline), growth hormone (GH)-receptor antagonists (pegvisomant) (7), and novel radiotherapy techniques.

Goals of radiotherapy include biochemical control and limiting tumor growth after inadequate response from surgery and/or medications (1, 8). Overall, radiotherapy is considered (1, 7) a third-line acromegaly treatment, in most countries; and a second-line option reserved for a patient-subset (1, 7). There is no comparative data on the effectiveness of radiotherapy vs medical therapy in patients with acromegaly and involving patients’ preferences for all adjuvant treatments decisions is paramount.

This opinion-piece discusses radiotherapy in the treatment paradigm, with a focus on outcomes of modern radiotherapy types.

## Types

### 1. Conventional radiotherapy

Conventional radiotherapy is administrated at a 40−45Gy dose delivered over at least 20 sessions. Although effective in biochemical normalization and controlling tumor growth, benefits are hampered by slow onset and high risk for adverse effects, e.g. hypopituitarism, optic neuropathy, and cerebrovascular disease (CVD) (9).

### 2. Modern stereotactic radiotherapy

Modern stereotactic radiotherapy techniques involve accurate localization in three dimensions, and is delivered either as single or multiple fractions. Major radiotherapy types utilized in most countries are stereotactic radiosurgery (SRS) and fractionated SRS (FSRT). Both deliver an accurate dose to a precisely defined target, with a steep dose gradient tumor margin, limiting damage to surrounding brain structure. Generally, the use of single-session SRS is limited to tumors less than 3cm in size and located more than 3mm away from optic structures, while FSRT is utilized in large pituitary tumors with optic nerve involved (9).

### 2a. Stereotactic radiosurgery

Stereotactic radiosurgery is typically applied in a single session; subtypes include gamma knife (GK), cyberknife, linear accelerator (LINAC) or proton-beam radiotherapy. Gamma knife is the most widely used radiotherapy type in treating pituitary adenomas (1, 8). Cyberknife image-guided robotic radiosurgery involves a LINAC mounted on a robotic arm; use is favored in patients with cavernous sinus invasion (10). Hypofractionated stereotactic radiosurgery uses 3−5 fractions for adenomas that are unsuitable for single session SRS treatment (9). Whole-sella SRS, targeting the entire sellar (between both cavernous sinuses) is an alternative approach (11). This approach is increasingly used for patients with functioning adenomas with irregularly shaped or radiologically indeterminate residual tumors. Whole-sella SRS has also been suggested for large tumors in patients with contraindications to surgery or intolerance to acromegaly medication. However, a recent study showed that whole-sella SRS is associated with higher rates of new visual deficit and mortality compared to targeted SRS. Though the difference was not statistically significant, based on the trend we consider that targeted SRS is favorable over whole-sella SRS (11).

### 2b. Fractionated stereotactic radiotherapy

Fractionated stereotactic radiotherapy (FSRT) is a technical improvement over CRT in which a total dose of 45−55Gy is delivered by a LINAC in 25−33 daily fractions. Immobilization in a frameless stereotactic mask and SRS-like planning allow better accuracy when compared to CRT (12).

## Efficacy

### Stereotactic radiosurgery

A meta-analysis including 20 published studies examined the effectiveness of SRS in treating acromegaly. The definition of endocrine remission (ER) varied across studies but was generally defined as a post-SRS GH < 1−2ng/mL and/or normal IGF-1 level for sex and age without the use of suppressive medication. The definition of endocrine control (EC) was defined similar to ER but with the use of medical therapy for acromegaly (13, 14). The 5-year ER rate was 43% and 10-year was 57%, while 5-year and 10-year EC rate was 55% and 70% respectively. Tumor control was achieved in most (93%) patients at year 10. Rates of new pituitary dysfunction were 27% and visual toxicity after SRS was 3% (13). Another meta-analysis showed tumor control was 97%, ER 44% and any new hypopituitarism 17%, however, the criteria used for ER were not uniform across studies (15). Interestingly, a 2021 retrospective study reported similar outcomes (16). Of 23 patients treated with GK, 48% of patients had ER by year 7 and an additional 39% achieved EC. Local tumor growth was controlled in all patients, but 30.4% developed new hypopituitarism post-SRS (16).

### Fractionated stereotactic radiotherapy

Data related to efficacy and adverse effects of FSRT use in patients with acromegaly are limited. A prospective single-center cohort study defined EC as normal sex and age-specific IGF-1 with/without medical treatment and ER as normal IGF-1 without any medication for a minimum of 3 consecutive months. Remission rates were 25% at 5, 43% at 10 and 50% at 15 years, respectively. Endocrine control was achieved in 97% at study-end and tumor growth was controlled in all patients. Patients (39%) had at least 1 new pituitary deficiency after 72 months of mean follow-up; importantly, no visual toxicities were observed (17).

### Comparative SRS and FSRT data

Comparative data studies of SRS, CRT and FSRT used in treating acromegaly have been published and summarized (9). Tumor control was achieved in 97−98% of patients for all three modalities.

Hormonal remission (median time to remission) was reached in 44% for SRS (41.5 months), 56% for CRT (73 months) and 35% (57 months) for FSRT. Stereotactic radiosurgery and FSRT led to new hypopituitarism in 22% and 29% and visual deficits in 1.5% and 1.8% of patients, respectively. Brain radionecrosis was 2.5% for CRT and < 1% for SRS and FSRT; CVD were observed in 20% for CRT, 4.5% for FSRT, and 0.3% for SRS (9). A single registry retrospective analysis compared FSRT (13 years) vs SRS (9 years) outcomes. Notably, GH levels pre-radiotherapy were higher in the FSRT group, implying more severe acromegaly. Remission was achieved in 48% of FSRT and 52% of SRS patients after 10 years and 71% and 78% in either remission or control at 10 years. Notably, mean time for EC was 3 years for FSRT and 2 years for SRS. Furthermore, there were few hypopituitarism differences; 80% of the FSRT and 63% of the SRS group had at least one secondary pituitary deficiency at the final visit. However, this includes both baseline and post-radiotherapy hypopituitarism (18). As expected, SRS was limited in clinical practice to smaller tumor remnants with a longer distance to the optic pathways, while FSRT was mainly used for larger, bilateral tumor remnants. Thus, patients who underwent SRS likely had milder disease, while patients treated with FSRT had more resistant acromegaly (19). Due to the non-randomized mode of selection, no robust differences in efficacy *per se* can be highlighted.

## Toxicities

### Stroke and cognitive sequelae

Stroke risk and cognitive sequelae are important factors to consider when using CRT (20). In a study of 118 patients treated using GK; median SRS dose was 30Gy and 81% of pituitary adenomas had cavernous sinus invasion (19). Median time to death post-SRS was 102 months with a wide range (18-243 months); 10-year stroke risk in post-SRS patients was 2.3%, this is low overall and is similar to that of the general population for incidence of first stroke (2.21%). Loss of visual acuity was 5.8% in patients post either magnetic resonance image (MRI)-guided SRS or for what is now considered outdated, computed tomography-guided SRS. However, visual impairment rate for patients who had MRI-guided SRS was lower at 2.2% (19).

Cognitive function effects of SRS are less well described. A cross-sectional non-randomized study compared cognitive function in patients with acromegaly as exposed to GK therapy (n = 27) vs unexposed EC/ER by surgery and/or medications (n = 37) (21). Almost a quarter of patients exhibited at least one abnormal cognitive function, but there was no significant difference between the GK-exposed and unexposed group (21). This highlights that GK appears to have less long-term sequelae. However, the small sample size of SRS studies and lack of data on patients who have undergone FRST underscores the importance of further research and monitoring.

### Visual

Visual toxicity includes radiation-induced damage of optic nerves, optic chiasm, and cranial nerves within cavernous sinuses. Risk for visual toxicity is variable; 0−6% after cumulative doses of < 54Gy with CRT and 9.3% for SRS with a mean dose of 16Gy. Optic neuropathy was < 2% when dosing was < 8−10Gy. For FSRT, optic neuropathy was < 2% when doses of < 50Gy were delivered in fractions of < 1.8Gy (22). Multidisciplinary discussions with radiation, ophthalmology, neurosurgery, and neuroendocrinology are recommended for both patient selection and radiotherapy type, to minimize visual risks.

### Risk of a second brain tumor

A multicenter retrospective cohort study analysis of second brain tumor occurrence in 3679 patients included 996 patients with pituitary adenomas or craniopharyngiomas exposed to radiotherapy compared to 2683 controls (23). Second brain tumors were reported in 61 patients (30 (3.10%) in the irradiated group; n = 966, and 31 (1.16%) in the control group; n = 2683). For those exposed to radiotherapy, 5 were malignant and 25 were benign brain tumors. For controls, 2 were malignant and 29 were benign brain tumors. Risk ratio for those patients exposed to radiotherapy was 2.18 (95% CI 1.31–3.62, p < 0.0001). At 20 years, the cumulative probability of second brain tumor was 4% for those patients exposed to radiotherapy compared to 2.1% for controls. The median latency period post-radiotherapy completion was 8.3 years for malignant and 17.7 years for benign tumors. Overall, there is an increased risk for second tumors post-radiotherapy exposer albeit at a rate lower than previously reported (23).

### New hypopituitarism

New hypopituitarism rates post-SRS vary from 17−30% (13, 15, 16). A retrospective study examined radiation-tolerance of surrounding adenomas structure in 521 SRS treated patients with adenomas (24). Nonfunctioning adenoma status, younger age, higher margin dose and higher doses to the pituitary stalk and normal tissues were independent predictors of new hypopituitarism. The median dose to the pituitary stalk for new endocrinopathy was 10.7Gy in a single fraction (OR 1.77, 95% CI 1.17−2.68, p = 0.006) (24). Another retrospective study (224 patients) found that a greater distance between the center of pituitary gland and the center of SRS-target was an independent predictor of pituitary function preservation (OR 1.101, 95% CI 1.00−1.21, p = 0.05). Normal pituitary function was observed in 73% of patients at a distance of 10−15mm, 86% at 15−20mm, and 90% at 20−25mm, respectively (25). This provides additional information to help minimize hypopituitarism while delivering effective radiotherapy for tumor control.

## Role of radiation in aggressive genetic syndrome tumors

The efficacy of radiation on either biochemical or tumor control based on histological subtypes of somatotroph adenomas is unknown. However, individuals with aryl hydrocarbon receptor interacting peptide mutations and acromegaly have earlier onset of disease, larger tumor size, higher GH secretion and resistance to SRLs (26). In addition to surgery and medications, radiotherapy should be considered early in the course of disease. Another inheritable syndrome involving GH-hypersecretion is X-linked acrogigantism (X-LAG). Patients with X-LAG syndrome do not response well to radiotherapy; it is not clear why this would be the case (27). Therefore, radiotherapy may lead to adverse events, while providing limited benefits overall.

## Discussion

Due to delayed diagnosis, patients with acromegaly often have large, invasive adenomas at presentation that are not cured by surgery alone; patients will require multimodal adjuvant treatment (1, 2). Lower mortality rates in patients with acromegaly over the past decade speaks in our opinion to the advancements made in treatment modalities, including modern radiation (1, 8).

Stereotactic radiosurgery is now a crucial armamentarium therapy and appears to be an effective and safe option for patients with acromegaly who have experienced unsuccessful surgery and have not responded to nor tolerated pharmaceutical agents. Selected patients with growing or aggressive tumors in the context of a genetic syndrome may need radiation even if biochemical control is achieved.

However, hypopituitarism is quite frequent and requires lifelong evaluation, as well as appropriate replacement therapy as hypopituitarism also increases mortality (28).

Desire for fertility should likewise be considered when discussing radiotherapy, given the high risk of hypopituitarism, and central hypogonadism. Counseling patients regarding fertility preservation is paramount even with new radiation techniques and improved fertilization *in vitro* methods. If there are larger residual tumors post-surgery and radiation is needed, pregnancy in the first 1−2 years after the radiation, but *after* achieving biochemical control of acromegaly with medical therapy, could be an option in the absence of visual changes and if the tumor is not close to optic chiasm. Notably, no medications to treat acromegaly are approved for use in pregnancy. However, there are SRL studies that report SRLs are relatively safe to continue until pregnancy is confirmed and can be restarted if needed during pregnancy (29). Many patients will not require treatment during pregnancy, as IGF-1 may be normal even after stopping therapy, however, after delivery IGF-1 can increase quickly and close follow-up care and observation is needed even if patients have undergone radiation in the past (2).

Studies examining the long-term cognitive effects of SRS treatment and patient-reported outcomes are warranted.

## Future

Further advancements in radiotherapy may impact the treatment paradigm in the future. Nevertheless, radiotherapy remains a third-line treatment after surgery and medical therapy for most patients. The role of radiotherapy in a patients treatment algorithm may need to be personalized based on modalities available per individual country. Patients with acromegaly should be managed in specialized centers to facilitate multidisciplinary decision making. This will also allow for joint physician-patient assessments of various modalities, that frequently multimodal treatments including surgery, medical therapy, and radiotherapy.

## Author contributions

Lead author WL drafted the manuscript and prepared the opinion. Author MF assisted with opinion preparation and review. WL and MF approved final opinion.
